# An interdisciplinary team-training protocol for robotic gynecologic surgery improves operating time and costs: analysis of a 4-year experience in a university hospital setting

**DOI:** 10.1007/s11701-021-01209-4

**Published:** 2021-02-19

**Authors:** Francesco Vigo, Rosalind Egg, Adreas Schoetzau, Celine Montavon, Midhat Brezak, Viola Heinzelmann-Schwarz, Tilemachos Kavvadias

**Affiliations:** Present Address: Department of Gynecology and Gynecologic Oncology, University Hospital of Basel, Spitalstrasse 21, 4031 Basel, Switzerland

**Keywords:** Robotic surgery, Gynecologic surgery, Surgical training, Advanced nurse practitioner

## Abstract

Main aim of this study is to assess the effect of a structured, interdisciplinary, surgical, team-training protocol in robotic gynecologic surgery, with the gradual integration of an advanced nurse practitioner. Data from all robotic surgical procedures were prospectively acquired. The surgical team consisted of one experienced surgeon and two surgical fellows and the scrub nurse team from three advance nurse practitioners, specialized in robotic surgery. The training was performed in a four-phase manner over 4 years and included theoretical training, hands-on training and team-communication skills enhancement. Scrub nurses increasingly adopted an active role during surgery. For a period of 4 years, 175 patients could be included in the analysis. All of them underwent a robotic gynecologic procedure. Mean docking time decreased from 45.3 to 27.3 min (*p* < 0.001), mean operating time from 235 to 179 min (*p* = 0.0071) and costs per case from 17,891 to 14,731 Swiss Francs (*p* = 0.035). There were no statistically significant changes in perioperative complications and conversions to laparotomy. An interdisciplinary long-term training protocol for high specialized robotic surgery within a “fixed” team with the gradually addition of an advanced study nurse improves the efficacy of the procedure in terms of time and costs. Although the surgery is performed quicker, the same performance and quality of surgical care could be reached.

## Introduction

Robotic-assisted laparoscopic surgery is the latest major development in minimally invasive gynecologic surgery. The Da Vinci robotic system was developed by Intuitive Surgical and was approved for gynecologic surgery by the United States Federal Drug Administration (FDA) in April 2005 [1]. Many advanced laparoscopic surgical interventions that would have been otherwise performed with an abdominal incision are now being performed with minimally invasive techniques utilizing this system [2].

Robotic surgery seems to have a steeper learning curve than conventional laparoscopy and demonstrates a faster adoption rate [3, 4]. It has been estimated that the overall proportion of cases performed through minimally invasive surgery has increased from 9 to 36% in the third year after introducing the robot and there is evidence that during the period 2013–2014 over 50% of all hysterectomies for benign indications in the United States were performed with the robot [5, 6]. Among others, this has been attributed to the fact that the robotic approach provides similar outcomes to conventional laparoscopy in terms of patient safety and efficacy while minimizing morbidity, postoperative pain, blood loss and hospital stay [7].

However, after an initial period of enthusiastic reception and commercial increment, robotic surgery has often been a target for criticism, mostly due to the long operating time and associated health costs [8]. Published data suggests that the costs of robotic-assisted hysterectomies can be up to 1.5–3 times higher than the costs of conventional laparoscopic techniques, despite overall shorter hospital stay and lower conversion rates [9]. This assumption, however, derives either from retrospective data or from publications, which did not provide appropriate economic evaluation (such as cost-minimization analysis, cost-effectiveness analysis, cost–benefit analysis and cost–utility analysis) [10, 11].

Structured and continuous training of young surgeons and surgeons with no robotic experience is crucial for the incorporation of robotic methods in clinical practice and maintenance of expertise, but it has also led to a re-thinking of training and teaching methods, not only for surgeons but for the entire operating room team too, including scrub and circulating nurses [12, 13]. Published studies have shown the advantages of highly trained personnel in the operating room but also the challenges and special requirements of continuous and state-of-the-art, multidisciplinary training in the operating room [14]. Also, since robotic surgery costs are generated in the operating room, there is evidence that robotic training and gain of surgical experience and expertise within the surgical team can lead to significantly shorter operating times and thus to eventually lower costs, even when compared to conventional techniques [15, 16].

Aim of this prospective study is to assess the impact and feasibility of a structured robotic surgery training protocol for gynecologic surgeons with the active integration of an Advanced Nurse Practitioner (ANP) and to present our experience in a university hospital setting.

## Materials and methods

This study, which obtained the approval of the local ethical committee (ID 355/11), was performed in the Department of Gynecology and Gynecologic Oncology of the University Hospital of Basel, and is a part of a multidisciplinary project to assess and support the implementation of robotic surgery as well as the employment of Advanced Nurse Practitioners, who are strategically employed in sensitive positions, to promote quality and patients’ safety. Our department began with the use of the four-arm da Vinci Si robot (da Vinci Surgical System ©, Intuitive Surgical Inc., Sunnyvale, CA, USA) on January 2012 for cases of gynecologic malignant and benign disease. A gynecologic/oncologic surgeon, with long-term experience in robotic surgery (VH) initiated the use of the robotic system. After the first period of acquaintance with the team and the setting, it was decided to begin with the implementation of the training protocol to expand and support the use of the system.

**Fig. 1:**
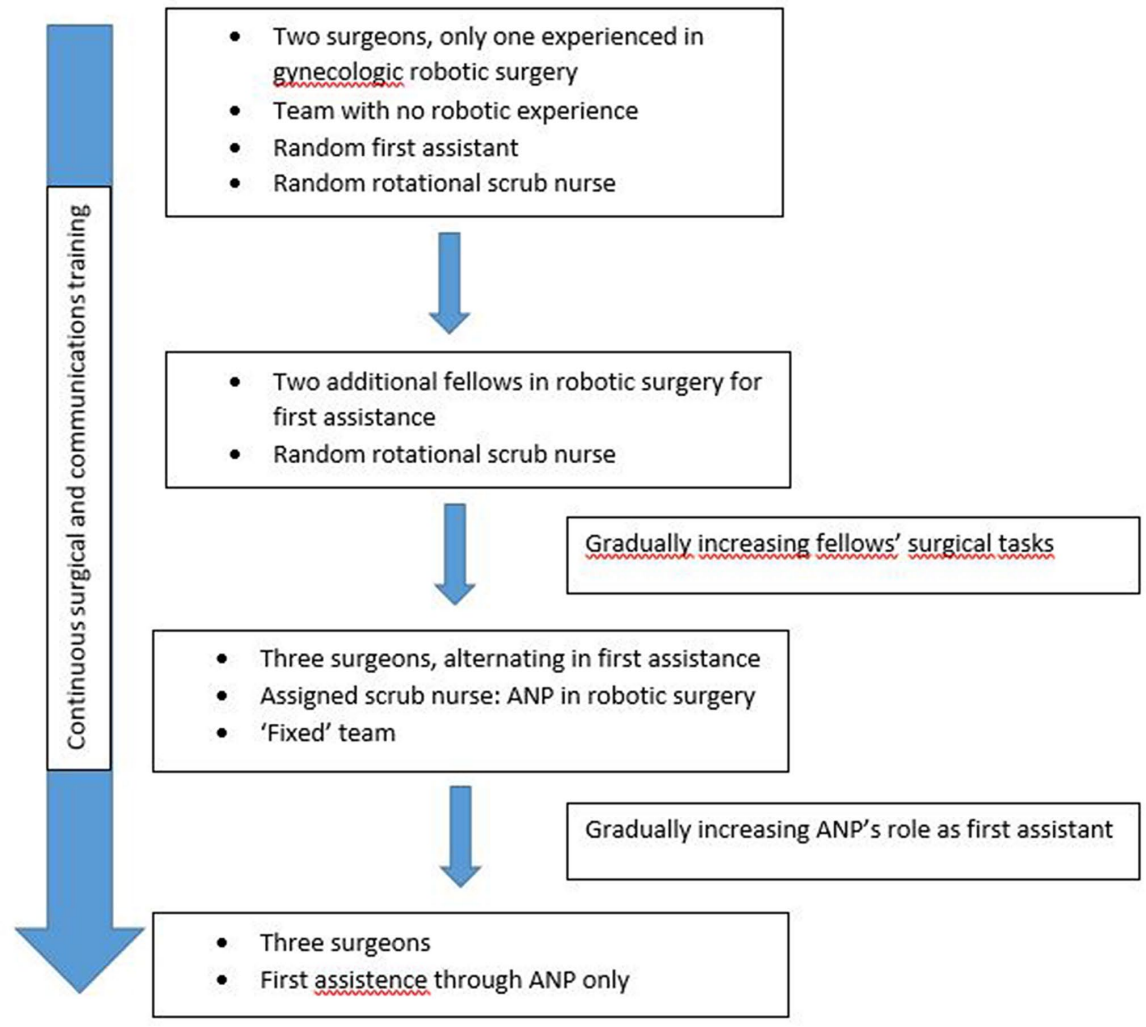
Graphical presentation of the training workflow

Aim of our training protocol aimed was the long-term, gradual reduction of the number of surgeons while simultaneously taking advantage of the acquired experience of the staff in robotic surgery. Hence, the implementation of the protocol corresponds to the outset of robotic surgery in our department. Since there was no previous experience with robotic surgery within the team, we decided to conduct a four-step-approach, which can be summarized as follows (see also Fig. 1):

Step 1 (baseline, time group 1): Initiation with a gynecologic/oncologic surgeon, with long experience in robotic surgery. Other team-members with no experience in robotic surgery. Random assigned first assistance as well as scrub and circulating nurse.

Step 2 (time group 2): Addition of two robotic surgery fellows (TK, CM) to the team, who alternately provided first assistance to the experienced surgeon. The two fellows received throughout a rigorous and continuous training including theoretical training, hands-on workshops and surgical training in robotic training centers in Europe as well as in-team feedback for their performance and expectations. The two fellows started having console time, starting with small tasks and gradually taking over whole steps of the surgical procedure. Scrub and circulating nurses were still randomly assigned.

Step 3 (time group 3): The two fellows are now fully integrated as surgeons to the team and the three surgeons were alternately performing, with one other providing first assistance. Thus, at any given time two experienced surgeons were present. Additionally, the scrub nurse team was reduced to three ‘fixed’ team members, who alternated places in preparing the robotic arms, performing intraoperative corrections and handing surgical instruments. Simultaneously, they received continuous theoretical and hands-on training with the surgeons. Gradually, integration of the scrub nurse as the first assistant, including instrumentation, suction, real-time correction of the robotic arms and active suturing assistance.

Step 4 (time group 4): Three surgeons (same as step 3). Addition of two ANP, who, alternately, completely took over the first assistance. At any given time only one out of three surgeons and one out of two ANP were by the patient. ANP conducts preparation, controlling and corrections of the robotic system and has full responsibility for the execution of the tasks at the patients while the surgeon sits at the console.

During this time, the entire team (surgeons and nurses) received continuous education and training on robotic surgery. The programme included three main educational domains:

1. theoretical training on laparoscopic surgery and operating room settings (anatomy, functional characteristics of the robot, regular literature updates),
2. intensive hands-on training (courses on robotic surgery, international workshops, simulation of emergency scenarios, training on cadaver and pelvic models) and
3. communications training (team time-out and sign-out, regular meetings, de-briefings and feedback after difficult situations, mutual decisions for surgery planning, joint social activities).

We collected data of all patients who underwent robotic surgery for malignant and benign gynecologic disease between January 2012 and December 2015. The operating time was defined as the time between skin incision and skin closure (at the trocar area), whereas the docking time was defined as the time between the first skin incision and console start with the surgeon in place and operating the robot. All procedures were performed according to our intern clinical practice protocols and in accordance with national recommendations, whereas especially in oncologic patients, the international requirements of clinical practice in oncology were applied. Patients with the diagnosis ovarian cancer in our analysis did not include the first diagnosis of ovarian, since in these cases the primary procedure is laparotomy, but it refers to patients who received hysterectomy or lymph node excision after the initial diagnosis was made.

This study was per protocol designed to conduct an analysis of perioperative data (such as docking and operating time) adverse events and costs, which were generated in the operating room and during the hospital stay, excluding costs from readmissions or outpatient treatments. Data was retrieved prospectively with the assistance of the hospital’s administrative and financial department.

### Statistical analysis

Descriptive statistics are presented as counts and frequencies for categorical data and median [interquantile range] for metric variables. Overall *p* values correspond to Kruskall–Wallis test (for median) and chi-squared or exact Fisher’s test when the expected frequencies is less than 5 in some cell.

To compare docking time, operative time, console time, overall costs, op costs, linear regression was performed between time groups. Results are presented as differences of means with corresponding 95% confidence intervals and *p* values.

To compare time groups between a hospital stay and blood loss, Dunn’s test was performed and 95% confidence intervals of the differences of medians were presented [17]. Confidence intervals were based on bootstrap. Dunn's test was performed because data distributions are considered as skewed. *p* values were considered as exploratory and not adjusted for multiple comparisons. A *p* value < 0.05 is considered as significant.

All evaluations were done using the statistical software R v 3.6.1 [18].

## Results

**Fig. 2:**
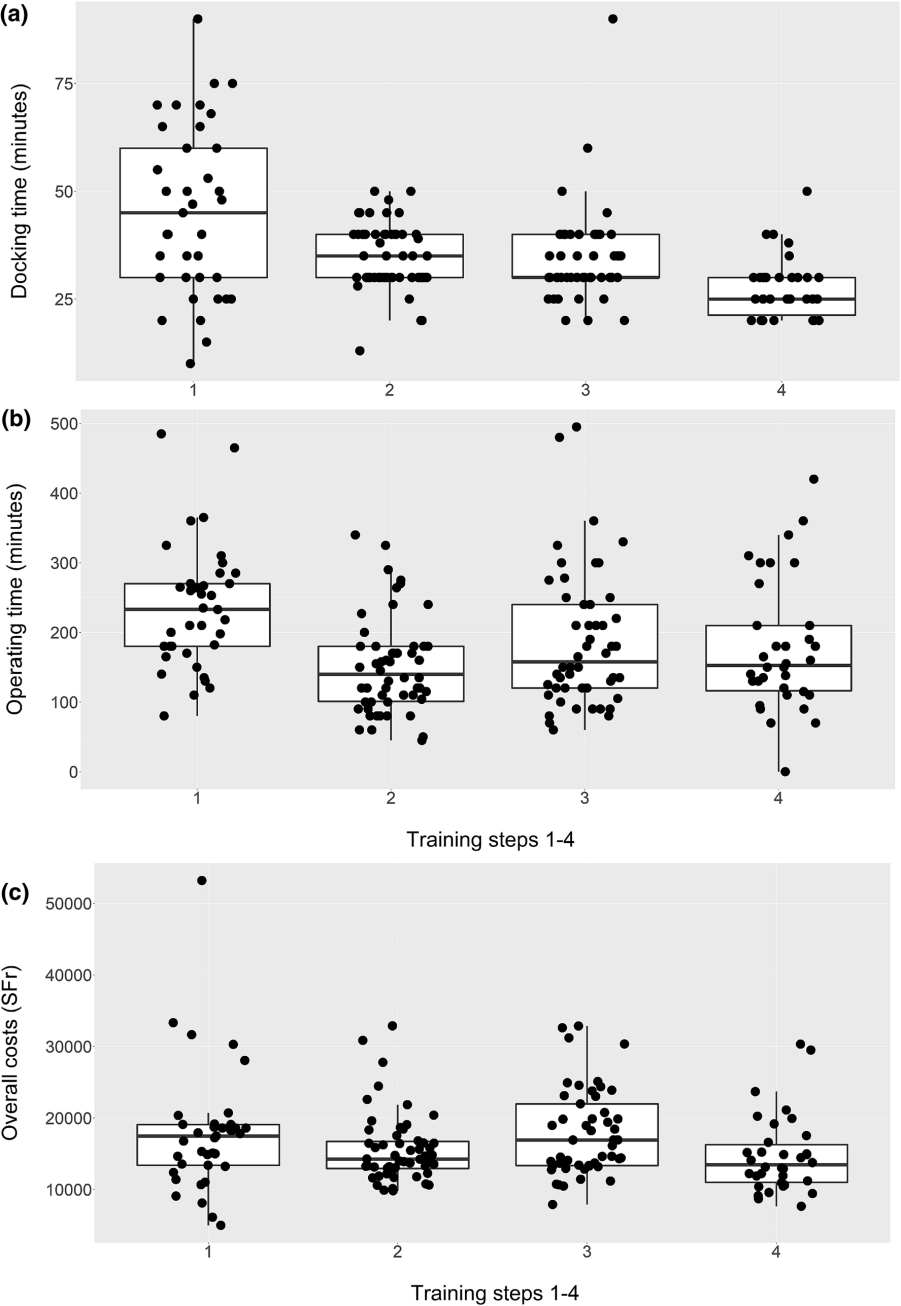
**a**–**c** Docking time, operating time and overall costs per case over time

We could include 175 robotic laparoscopic procedures, which were performed in the department of gynecology and gynecologic oncology between January 2012 and December 2015. Table 1 shows number of patients included in each step as well as patients’ characteristics and main indications. Between time group 1 and time group 4, mean docking time decreased significantly from 45.3 to 27.3 min (mean difference: 18; CI 12–24 *p* < 0.001), mean operative time decreased from 235 to 179 min (mean difference: 57; CI 16–98; *p* = 0.0071. The mean costs per case generated in the operating room were reduced from 6907 to 6506 (mean difference: 402; CI − 758 to 1562; *p* = 0.50) but were not significant. The mean overall costs per case decreased significantly, from 17,891 to 14,731 Swiss Francs (mean difference: 3159; CI 213–6105; *p* = 0.035). Median hospital stay decreased not significantly from 4 to 3 days (median difference: 1; CI 0–2 *p* = 0.14). There were no statistically significant changes in perioperative complications and conversions to laparotomy (8.11% vs. 3.00%, *p* = 0.457 and 8.11% vs. 5.88%, *p* = 0.390, respectively). Also, there were no statistically significant differences in intraoperative complications and conversions to laparotomy. Overall median blood loss was significantly higher at the end of the study period (100 ml vs. 150 ml, median difference: − 50, CI − 123 to 25, *p* = 0.012) (for detailed information please see Table 2, as well as Fig. 2a–c).

**Table 1:** Patients’ characteristics and diagnoses

Patient characteristics	All (*N* = 175)	Phase 1 (*N* = 37)	Phase 2 (*N* = 54)	Phase 3 (*N* = 50)	Phase 4 (*N* = 34)	
Age (medan, IQR)	47.6 [42.4; 56.2]	44.9 [39.2; 50.6]	49.5 [42.2; 59.7]	48.5 [42.7; 64.2]	49.8 [45.5; 54.7]	*p* = 0.098
BMI (median, IQR)	27.0 [23.0; 31.0]	27.0 [23.0; 29.0]	27.0 [22.2; 34.4]	26.5 [23.0; 31.8]	25.0 [22.2; 29.8]	*p* = 0.745
Previous surgery	76 (43%)	16 (43%)	25 (46%)	23 (46%)	12 (36%)	*p* = 0.45
CCI (*N*)						*p* = 0.305
0 1 2 3 +	108 11 37 18	23 5 7 2	32 3 13 6	32 2 13 8	18 1 5 2	
Diagnoses						*p* = 0.65
Leiomyoma Endometrial cancer Cervical cancer Pelvic organ prolapse Ovarian cancer Other^a^	94 46 11 7 5 12	22 6 3 3 1 2	27 17 2 3 2 2	26 17 4 1 2 8	19 6 1 0 0 0	

*CCI* Charlsson Comorbidity Index, *IQR* interquartile range

^a^Other diagnoses include: endometriosis, benign ovarian mass, gender dysphoria, elective ovarectomy and persistent cervical dysplasia

**Table 2:** Surgical interventions and intraoperative data

	All	Phase 1 (*N* = 37)	Phase 2 (*N* = 54)	Phase 3 (*N* = 50)	Phase 4 (*N* = 34)	
Interventions						*p* = 0.187
Hysterectomy Leiomyomectomy Adnexectomy Pelvic node removal Sacrocolpopexy	119 36 67 24 6	18 12 9 4 3	38 10 22 3 2	24 11 29 13 1	24 3 7 4 0	
Operating time (median, IQR)	165 [120; 240]	233 [180; 270]	140 [101; 180]	158 [120; 240]	152 [116; 210]	*p* < 0.0001
Docking time (median, IQR)	30.0 [30.0; 40.0]	45.0 [30.0; 60.0]	35.0 [30.0; 40.0]	30.0 [30.0; 40.0]	25.0 [21.2; 30.0]	*p* = 0.016
Complications (intraoperative)	15 (8.67%)	3 (8.11%)	4 (7.69%)	7 (14.0%)	1 (3.00%)	*p* = 0.390
Laparotomy conversions	10 (5.71%)	3 (8.11%)	4 (5.6%)	4 (8.00%)	2 (5.88%)	*p* = 0.457
Mean blood loss	100 [50; 200]	100 [50.0; 200]	50.0 [20.0; 188]	100 [50.0; 200]	150 [100; 288]	*p* = 0.011

*IQR* interquartile range

## Discussion

This prospective study presents the impact of a structured educational protocol on the effectiveness of a team of both young surgeons and specialized nurses. To our knowledge, this is the first prospective analysis of such an effort, which includes the education of medical doctors as surgical fellows and simultaneously the introduction of the Advanced Nurse Practitioner as constant team members in robotic surgery, with an active role during the surgical intervention. Our strategic goal was to increase the number of experienced robotic surgeons in our team, without compromising quality and patients’ care, since the growing demand on minimally invasive surgery and novel technologies dictated the investment on and the further development of robotic surgery in gynecological patients of our department.

The importance of continuous surgical training and frequent surgical activity in the operating room in achieving and maintaining surgical excellence and improving perioperative patients’ care is undeniable [19, 20]. In addition, education and training of young surgeons in an out of the operating room is crucial for gaining surgical proficiency, mostly so in complex interventions [21]. A systematic review of Miskovic et al. reported that trainees could obtain clinical results similar to those of expert surgeons in laparoscopic colorectal surgery if mentored and supervised by an experienced trainer [22]. They showed that a structured training of young surgeons on laparoscopic colorectal intervention with a mentor assignment, results in similar conversion rates (*p* = 0.28), complications (*p* = 0.49), anastomosis leak (*p* = 0.36) and mortality (*p* = 0.56), when compared to interventions performed by experts. However, there is evidence that due to variable factors, such as novel non-interventional therapies and the expansion of radiological-interventional methods, residents and trainees can suffer from the lack of proper surgical training, much more so in advanced and not-so-frequent performed surgical interventions [23].

Robotic surgery can be definitely seen as a ‘special case’ as far as surgical training is concerned. The vast majority of surgical interventions are performed with one console and there is one principal surgeon, controlling the robotic arms and the camera. The physical distance between the operating surgeon and operative field creates a barrier that prevents a resident learner from appreciating how an attending's physical movements directly translate into the simultaneous tissue manipulation observed on the screen [24]. The learner often struggles to recreate these movements, while other physical barriers, such as screen size, arm placement and the lack of a haptic feedback may compromise the young surgeon’s experience and also decrease the trainers consent to give over control [25].

With the increasing demand and use of robotic systems, residents’ and OR team training became profoundly important for patients’ safety and quality assurance. The development of a structured training curriculum in robotic surgery has been proposed as the major prerequisite for the successful transfer of skills to young surgeons from their experienced mentors [26]. As mentioned before, this is not an easy task in the case of robotic surgery, but there is evidence that in a proper setting, the training of young surgeons can be feasible, efficient and safe [27].

Similarly important, although not as thoroughly examined, seems to be the training of the operating room nurse. Similar to medical trainees, nurses who work with robotic systems, are confronted with a number of conceptual and technical challenges. First, two of the most important behavioral markers for successful nursing in the operating room, namely the eye gaze/contact with the surgeon and the anticipation movements, are compromised, due to the fact, that the surgeon sits alone at the console [28]. Second, robotic surgery demands high technical competence and more active role from the operating room nurse, whose responsibilities involve helping the surgeon, paying attention to the rules of asepsis by distinguishing the sterile and nonsterile parts of the robot, placing the robot arms, reading the data received from the videoscopic screen correctly and quickly, reporting to the surgeon and taking immediate measures in case of possible power failure [29, 30]. Consequently, these demands require a comprehensive and effective training of the robotic nurses, to maintain quality but also to overcome individual fear and hesitation when dealing with the robotic system [31].

It can be argued that the conventional model of a surgical curriculum is mainly single surgeon oriented and hierarchical structured, lacking interdisciplinary involvement, rather impersonal and mostly short-term regarding team- and professional development of the people involved. The approach in this study focused on interprofessional interaction and awareness, team strengthening measures, continuous medical and technical training and long-term planning and objective targeting.

Of course, the improved outcomes could partially be explained through the gained experience of the surgeon and simply the comfort of all participants with the robotic technique and there is evidence that surgical team training, including scrub and circulating nurses can improve outcomes [32]. Our data, being to our knowledge the first publication which includes the active integration of an advanced nurse practitioner in the role of the first assistant, show that a structured training protocol based on the increasingly active role of operating room nurses and novice fellow-surgeons is not only feasible but also beneficial in terms of patients’ safety, perioperative time and generated costs. Outcomes like improved docking time, which continuously decreased while performed by different team members throughout the different phases, support the fact that team training and active involvement of everyone present in the operating room, and not only the main surgeon, is crucial. This could be shown in a prospective manner, including all gynecologic patients who were treated with robotic surgery. Our results also showed an increase in blood loss between the first and the last training phase. Blood loss estimation in our study was performed empirically through the surgical team, not on the basis of absolute quantitative methods, such as fluid balance in the suction device or gauze weighing. This fact alone could present a strong bias for the estimated blood loss. Additionally, in step 4—in contrast to step 3—the first assistance was performed solely through the ANP, so there might have been a significant variability regarding suction, irrigation, etc. which could affect the estimation of blood loss. However, the small difference in the mean blood loss (50 ml) is not of clinical significance. This is also indicated from the not significantly different complications rate, including the need for blood transfusion.

Main limitations of this study include the lack of a control group and the fact that our starting point was very early in the adoption process of robotic surgery in our institution, which could be factors that limit the generalization of the results. Further limitations of this study is the small sample size (mostly in phase 1 and 4) as well as the lack of a power analysis.

## Conclusion

Although often criticized, robotic surgery is still gaining popularity and the number of robotic procedures is rising. Not only medical but also nursing personnel face important technical and conceptual challenges when using robotic surgical systems. Multidisciplinary, continuous and structured training should be offered to all teams working with robotic systems in order to maintain quality and improve skills and technical competence.

## Data Availability

Data and source documents are available for review through the local ethics committee.
